# EphB2 Deficiency Induces Depression-Like Behaviors and Memory Impairment: Involvement of NMDA 2B Receptor Dependent Signaling

**DOI:** 10.3389/fphar.2018.00862

**Published:** 2018-08-07

**Authors:** Linlin Zhen, Tuo Shao, Victor Luria, Gaowen Li, Zhi Li, Ying Xu, Xin Zhao

**Affiliations:** ^1^Department of Breast and Thyroid Surgery, The Affiliated Huai'an No. 1 People's Hospital of Nanjing Medical University, Huai'an, China; ^2^Brain Institute, School of Pharmacy, Wenzhou Medical University, Wenzhou, China; ^3^Department of Systems Biology, Harvard University Medical School, Boston, MA, United States; ^4^Department of Pharmacology, Ningbo College of Health Sciences, Ningbo, China; ^5^School of Pharmacy and Pharmaceutical Sciences, University at Buffalo, State University of New York, Buffalo, NY, United States; ^6^Department of Pharmacology, School of Medical Science, Ningbo University, Ningbo, China

**Keywords:** EphB2, depression, cognition, neurogenesis, NMDA-2B receptor, NR2B antagonist Ro25-6981

## Abstract

Receptor tyrosine kinase EphB2 mediates development of the neurogenic niche of excitatory neurons, suggesting the possibility that its inactivation plays a role in neuropsychiatric disorders including depression and memory impairment. While N-methyl-D-aspartate (NMDA) receptor is involved in regulating memory formation and neurogenesis in adult animal, it remains unclear how NMDA receptor subtypes mediate depression and cognitive deficits caused by EphB2 loss. The present study shows that EphB2 inactivation results in depression-like behaviors, memory impairment and defects of adult hippocampal neurogenesis. Compared to wild-type littermates, EphB2 KO mice exhibited depression-like behavior and deficits in spatial memory and cognition in forced swimming, tail suspension, Morris water maze, object recognition test and object location test. These behavioral abnormalities were accompanied by substantial decreases in the number of BrdU+ progenitor neurons, phosphorylation of cAMP-response element binding protein (pCREB) and brain derived neurotrophic factor (BDNF), and increased NMDA receptor 2B (NR2B) expression. These molecular, cellular and behavioral alterations induced by EphB2 inactivation were reversed by NR2B antagonist Ro25-6981, suggesting that EphB2 functions to prevent the progression of depression-like behavior and memory impairment by downregulating NR2B. Our findings highlight that NR2B is responsible for EphB2-dependent behavioral and morphological changes. EphB2 may thus be as an important candidate target for treating psychiatric and cognitive disorders.

## Introduction

The Eph family of receptor tyrosine kinases is subdivided into EphA and EphB receptors, based on their ability to bind two subclasses of cognate ligands, ephrinAs or ephrinBs, respectively (Himanen and Nikolov, 2003). EphA and EphB receptors and ephrin ligands function to regulate the organization and connectivity of the developing nervous system in many organisms (Cramer and Miko, 2016). At least four members of the Eph receptor family including EphA4, EphB1, EphB2, and EphB3 are highly expressed in the prefrontal cortex, hippocampus and amygdala, whose functions are closely related to emotional and cognitive disorders, such as depression, anxiety, and memory deficits (Liebl et al., 2003). Previous studies indicated that tyrosine phosphorylation of EphB2 is required for hippocampal long-term potentiation (LTP) and spatial learning and memory (Bouzioukh et al., 2007; Cissé et al., 2011; Hu et al., 2017). Moreover, activation of EphB2 receptor protects animals against chronic stress-induced depressant-like behaviors (Zhang et al., 2016), which indicates a major role of EphB2 in psychiatric disorders. Indeed, depression is generally accompanied by cognitive and memory deficits, which are critical determinants of functional outcomes in patients with major depressive disorders (Jaeger et al., 2006). Nevertheless, it is not well understood how does loss of EphB2 mediate depression and associated cognitive deficits.

Persistent neurogenesis has been consistently observed in the adult mammalian brain in the subgranular zone of the dentate gyrus (DG) and the subventricular zone (SVZ) of the lateral ventricles in the hippocampus (Kiselycznyk et al., 2015). In the adult dentate gyrus, newborn neurons are generated from a pool of neural progenitor cells before maturing and integrating into the hippocampal circuitry (Xu et al., 2007; Hu et al., 2008). Hippocampal neurogenesis is required for antidepressant-like behaviors and for memory enhancing effects in some circumstances (Xu et al., 2007; Li et al., 2009). Our previous studies suggested that chronic stress-induced decreases in hippocampal neurogenesis and neuronal atrophy may result from upregulation of NR2B and related downregulation of brain derived neurotrophic factor (BDNF) levels (Xu et al., 2007, 2009). As a transmembrane tyrosine-kinase receptor, EphB2 mediates tyrosine phosphorylation of NMDA receptor subunits and regulates the synaptic localization of NMDA receptors. Recent findings show that EphB1, EphB2, and EphB3 KO mice exhibit homeostatic upregulation of NMDAR surface expression (Xia et al., 2014). Further studies demonstrated that EphB-NMDA receptor interaction in cortical neurons regulates synaptic remodeling and emotional responses such as neuropathic pain and cognitive deficits (Shi et al., 2016; Hanamura et al., 2017). Among the signals that regulate the architecture of the neurogenic niche, the EphB2 receptor and its ligands are important by directly interacting with N-methyl-D-aspartate receptors to stimulate neurogenesis. Since depletion of EphB2 results in a reduction of functional synaptically localized NMDARs and in a reduction of long-term potentiation (LTP), it is possible that knockout of EphB2 induces neuronal dysfunction. Nevertheless, it has remained unclear whether the emotional alterations of EphB2 knockout mice are related to NMDAR subunits.

In this study we demonstrate that the mutant mice expressing a truncated form of EphB2 that lacks the entire functional domain (Henkemeyer et al., 2003) display abnormalities in behavior and hippocampal neurogenesis, altered expression of NR2B and its downstream signaling molecules, such as the phosphorylation of cAMP-response element binding protein (pCREB) and BDNF. We further show that pharmacological inhibition of NR2B by Ro25-6981 reversed EphB2 knockout-induced depression-like behaviors and associated memory impairment.

## Materials and methods

### Animals

The EphB2 KO mice were generated (Henkemeyer et al., 2003) and generously provided by Dr. Mark Henkemeyer of UT Southwestern. EphB2 KO animals and wild-type (WT) control littermates have the same genetic background (mixed CD1 and C57BL/6). They were bred and housed in the Animal Resource Center at the West Virginia University Health Sciences Center. All experiments on animals were conducted according to the “NIH Guide for the Care and Use of Laboratory Animals” (revised 2011) and were approved by the Institutional Animal Care and Use Committee at West Virginia University and Ningbo University.

### Reagents

Ro25-6981 hydrochloride hydrate was purchased from Sigma-Aldrich (Millipore Sigma, USA), and was administered 30 min (min) before behavioral tests. The following primary antibodies and dilutions were used for immunohistochemistry (IHC) staining: rat anti-BrdU IgG (1:1,000; Abcam), mouse monoclonal anti-GFAP (1:1,000; Millipore), mouse monoclonal anti-NeuN (1:200; Millipore), anti-rat FITC (1:200; Invitrogen, Carlsbad, CA), anti-mouse Rhodamine Red-X (1:200; Invitrogen), and anti-rabbit Cy5 (1:200; Invitrogen). The antibodies below were used for immunoblotting: mouse monoclonal anti-NR2B (1:1,000; Millipore), mouse monoclonal anti-BDNF (1:1,000; Millipore), rabbit monoclonal anti-phospho CREB and anti-CREB (both 1:1,000; Millipore) and β-actin (1:10,000; Abcam). Alexa Fluor 700 conjugated goat anti-rabbit or goat anti-mouse antibodies (both 1:20,000; Invitrogen, Eugene, OR) were used as secondary antibodies.

### Bromodeoxyuridine administration

Single intraperitoneal injections of Bromodeoxyuridine (BrdU, 150 mg/kg) were used to study proliferation (the first trial, T1) and differentiation (the second trial, T2) in the dentate gyrus (DG). BrdU (Sigma, St. Louis, MO) was dissolved in 7 mM NaOH in PBS (0.1 M phosphate-buffered saline at pH 7.4) to a final concentration of 16 mM (5 mg BrdU into 1 mL NaOH/PBS). The BrdU was shaken, heated to 37°C until dissolved and cooled down before being injected. In experiments with a single injection, animals were sacrificed 24 h (h) after BrdU administration (T1). In T2 experiments for staining with anti-BrdU and anti-NeuN, or double staining with anti-BrdU and anti-GFAP, animals were sacrificed 28 days after BrdU administration. Ten EphB2 KO mice and 10 WT littermates per group were used in both T1 and T2 experiments.

### Forced swimming and tail suspension tests (FST and TST)

These two tests are usually assessing depression-like behaviors by comparing the immobility time of animals in inescapable situations with those of untreated littermates. Eight-week old mice were subjected to a test in the parallel environment. In the forced swimming test (Porsolt et al., 1978), mice were placed in transparent beakers with 20 centimeters (cm) deep water at 23–25°C. Water was changed between subjects. The mouse was forced to keep swimming or remaining floating motionless in the water, to make sure it was not sinking. In the tail suspension test (Xu et al., 2005), mouse tail was taped at a bar 50 cm above the floor. The process of every cycle for both FST and TST was videotaped for 6 min (min) and the immobility time within the last 4 min was scored. The definition of “immobility” is as follows: freezing or just making necessary movements to keep balance in the water or in the air.

### Locomotor activity (LA)

Locomotor activity was determined in the open field test utilizing an automated activity monitoring system (San Diego Instruments, San Diego, CA). Mice were acclimated to the testing facility for at least 30 min and habituated to the testing chambers for an additional 30 min before locomotor activity analysis. Each testing chamber consisted of a Plexiglas housing and a 16 × 16 photobeam array to detect lateral (ambulatory and fine) movements, with a separate 16 photobeam array to detect rearing activity. Mice were treated and placed back in their respective chambers during the acclimation period. Ambulatory, fine and rearing movements were recorded and summated as a measure of total locomotor activity for the next 30 min. Ten EphB2 KO mice and 10 WT littermates per group were used in these TST and FST. Animals (10 mice/group) were sacrificed after locomotor activity test for subsequent immunoblots analyses, such as NR2B, pCREB, and BDNF expression.

### Morris water maze assay (MWM)

This test is used to assess the spatial memory of mice following the procedures described previously (Nunez, 2008) with minor modifications. The water maze consisted of a blue circular pool with a diameter of 1.2 meter (m) and depth of 76 cm, filled with 25–27°C water. Inside the pool a transparent platform (of 35 cm diameter) was placed 1.5 cm under the water level. A light source and patterns on the wall surrounding the pool served as extra maze cues. Mice were trained for six blocks consisting of three (60 s) trials separated by 20 min inter-block intervals (acquisition of learning). On each trial, the mice were placed in the water from different start location (East, South, West and North). The platform remained in the same location relative to the distal cues in the room during the training session. To measure the consolidation of learning and memory, 1 and 24 h after the sixth block of training, the hidden platform was removed and mice were scored during a 60 s probe trial for latency to and crossings over the previous platform location. The experimental procedures were recorded on a videotape and analyzed by the Ethovision XT Software (Noldus Information Technology, Wageningen, Netherlands).

### Novel object location test (OLT) and novel object recognition test (ORT)

A testing session comprises two 3 min trials according to the method described previously (Rutten et al., 2009). During the first training session (T1), two identical objects (samples) were put into the apparatus. A mouse was placed in the apparatus facing the wall in the center of the segment with the transparent front. The mouse was put back into its home cage after a 3-min exploration period. One and twenty-four hours after the T1 training session, the mouse was put back into the apparatus for the test session (T2). One object remained in its previous position, the other was placed in a novel location, which was 20 cm toward the front of the arena for the other object. In the ORT (T2), one of the original objects in T1 was replaced by a novel object (Ennaceur and Delacour, 1988). The time spent in exploring the object in the novel location and novel object was recorded automatically by the videotape that was connected with a computer. All combinations and locations of objects were used in a balanced design to reduce potential bias due to any preferences for particular locations or objects. Exploration was defined as follows: directing the nose to the object at a distance of no more than 2 cm and/or touching the object with the nose. Sitting on the object was not considered exploratory behavior. To avoid the presence of olfactory trails, the objects were thoroughly cleaned after each trial.

The experimenter was blind to the genotypic status of EphB2 KO mice and WT littermates. Data were analyzed by the Ethovision XT Software. A ratio of time spent exploring any one of the two objects in the training session, or the novel object (relocated object) in the retention session over the total time spent exploring both objects was measured as the preference cognitive index. Other cohorts of animals (10 mice/group) were chosen for memory and cognitive tests, the subsequent immunohistochemical and immunofluorescent analyses were performed after behavioral tests.

### Immunohistochemistry and immunofluorescence on mouse brain sections

Mice were anesthetized with ketamine/xylazine, perfused intracardially with ice-cold solutions of 10 mL of 0.9% physiological saline, and then followed by 10 mL of 4% paraformaldehyde (PFA) in PBS. Brains were removed and fixed in 4% PFA for 24 h at 4°C, after that in 30% sucrose (dissolved in PBS) overnight at 4°C, and then followed by 30% (w/v) sucrose/PBS at 4°C until brains sunk. Brains were then embedded in Tissue-Tek (Sakura) for 24 h at −20°C.

Serial sections (40 μm, 1 in 6 series) were cut through the entire hippocampus (−1.72 to −6.72 mm from the bregma, Paxinos and Watson, 2005) on a freezing microtome (Leica). Brain sections (18 sections from one brain, 180 sections from 10 brains) were washed once with 0.1 M PBS, pH 7.4, incubated in 2 × SSC/50% formaldehyde at 65°C for 2 h, followed by 2 N HCl at 37°C for 30 min and 0.1 M boric acid (pH 8.5) for 10 min before blocking with bovine serum albumin (BSA) for 30 min. Brain slices were incubated in primary antibodies overnight at 4 °C. Brain sections were then washed by PBST buffer (PBS with 1% Tween-20 detergent) for three times for 15 min followed by incubation in secondary antibodies dissolved in PBST buffer for 1 h at room temperature on a platform shaker. Unless otherwise stated, all immunohistochemistry (IHC) procedures used in the experiments were done at room temperature. Brain sections were then mounted on adhesion microscope slides. The BrdU+, NeuN+, and GFAP+ cells were visualized and counted using a Bio-Rad Radiance 2100 confocal system (Bio-Rad Laboratories, Inc., Hercules, CA) with a Nikon TE300 microscope (Nikon, Tokyo, Japan).

### Protein electrophoresis and western blotting

Mouse hippocampal tissues were lysed with RIPA lysis buffer containing protease and phosphatase inhibitors and centrifuged at 13,000 rpm for 30 min at 4°C. Total protein concentrations was determined in the supernatant using the BCA protein assay kit (Thermo Fisher Scientific, USA). Samples (30 μg) were separated using SDS-PAGE and then transferred to PVDF membranes. Membranes were then incubated with rabbit antisera against pCREB (Ser133), CREB, NR2B, BDNF (all at 1:1,000 dilution; Millipore) and β-actin (inner control; 1:10,000; Abcam). The next morning, membranes were washed then incubated with Alexa Fluor 700 conjugated goat anti-rabbit or goat anti-mouse antibodies (both 1:20,000; Invitrogen, Eugene, OR) for 30 min. The detection and quantification of specific bands were carried out using a fluorescence scanner (Odyssey Infrared Imaging System, LI-COR Biotechnology, Lincoln, NE). For band stripping, the membranes were incubated with stripping buffer (Thermo Fisher Scientific, USA) for 10 min.

### Stereology

All images were captured using a confocal laser scanning microscope (Zeiss LSM510, Thornwood, NY). For proliferation assays, BrdU-positive (BrdU^+^) cells in the sub-granular zone of the dentate gyrus were counted from the left side of every sixth cryosection (40 μm section thickness). Given the limited number of BrdU^+^ cells, all BrdU^+^ cells were counted except for positive cells in the uppermost focal plane, in order to avoid oversampling (Kempermann et al., 1997). To assess differentiation at 28 days, tissues were labeled for BrdU, NeuN, and GFAP. BrdU+ cells throughout the granular layer were counted from both sides of every sixth section and identified as neurons, astrocytes, or other by confocal microscopy.

### Statistical analyses

Data were expressed as the mean ± standard error of the mean (S.E.M.). The significance of differences between genotypic groups were analyzed by one-way analysis of variance followed by Bonferroni's *post-hoc* tests unless otherwise noted utilizing GraphPad Prism version 6.0 (San Diego, CA). A *P* < 0.05 was considered statistically significant.

## Results

### EphB2 KO mice exhibited depression-like behaviors in mouse models of despair test

**Figure 1:**
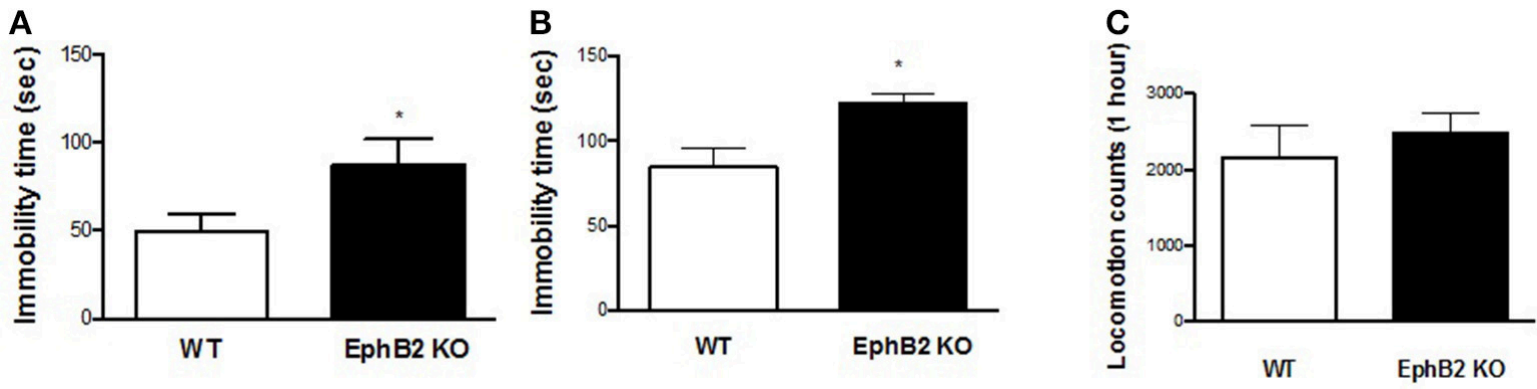
EphB2 deficiency (EphB2 knockout) induced depression-like behaviors. Immobility times both in the forced swimming and tail suspension tests increased **(A,B)**. Locomotor activity **(C)** did not change in the EphB2 KO mice. *P < 0.05, vs. the wild type littermates (WT).

The forced swimming and tail suspension tests (FST and TST) are two standardized paradigms for the assessment of despair behavior by analysing immobility time in inescapable aversive situations. In the FST, EphB2 KO mice started to float significantly earlier and showed longer total immobility time (Figure 1A) than wild-type (WT) littermates (*p* < 0.05). The increased immobility time in mutants was also observed in the tail suspension test, in which mutants had longer immobility times than wild-type controls (*p* < 0.05; Figure 1B). However, the locomotor counts did not show significant changes between EphB2 KO mice and their littermates as shown in Figure 1C.

### EphB2 deficiency-induced spatial learning and memory deficits in the morris water maze test

**Figure 2:**
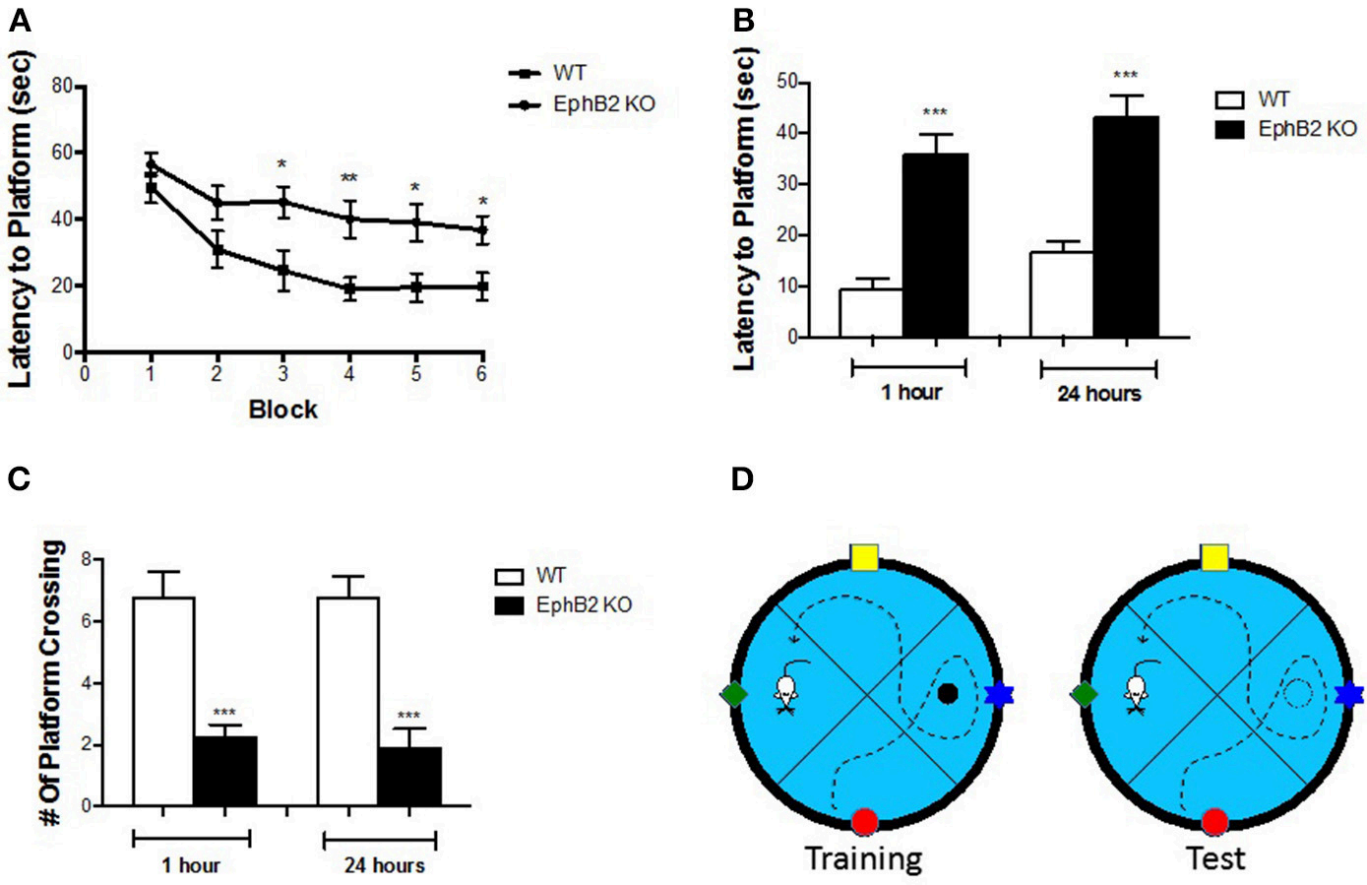
EphB2 deficiency induced spatial learning and memory deficits. **(A)** Learning curve in the water maze task for EphB2 KO and WT mice. **(B)** Latency to reach the platform during the 1 and 24 h probe trials in EphB2 KO and WT mice. **(C)** The number of crossing during the 1 and 24 h probe trials in EphB2 KO and WT mice. *P < 0.05, **P < 0.01, and ***P < 0.001, vs. the wild type group. **(D)** Schematics of the Morris water maze test.

One important function of the hippocampus is to control emotional behaviors, such as depression and anxiety, as well as defects in learning and memory performance. To test whether the loss of EphB2 affects spatial learning and memory, adult EphB2 KO mice and matched WT littermates were examined using the Morris water maze test (MWM) (Figure 2D). In the spatial reference training (visible platform) session, all groups of mice showed a reduction in latency (search time) over successive trials. The EphB2 KO mice spent a longer time to reach the platform than WT littermates from the 3, 4, 5, and 6th trial blocks (Figure 2A). In the hidden platform component of the test (which tests spatial memory), EphB2 KO mice did not retain a clear bias for the platform location 1 and 24 h after the last training session. The average time needed to locate the previous platform location was significantly increased in EphB2 KO mice, when compared to WT littermates (both *p* < 0.001 at 1 and 24 h after training; Figure 2B). The number of platform crossings to the target quadrant where the previous platform had been located in the training session was fewer in EphB2 KO mice than that of WT littermates, both at 1 h and 24 h after training session (*p*'s < 0.001; Figure 2C).

### EphB2 deficiency-induced cognitive deficits in the novel object recognition and location tests

**Figure 3:**
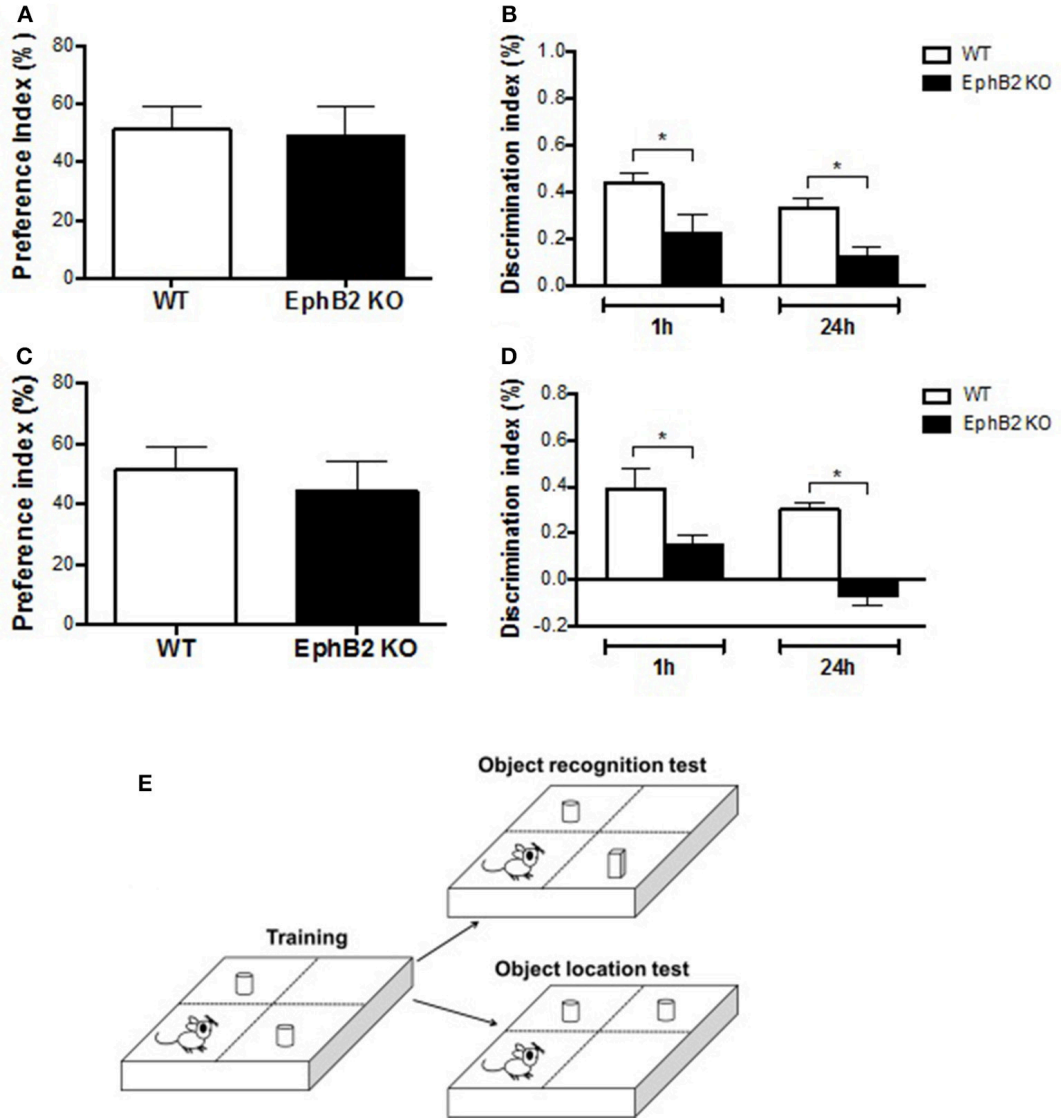
EphB2 deficiency induced cognitive impairment. The novel object recognition test **(A,B)** and novel object location test **(C,D)** were performed. In the training sessions **(A,C)**, there was no statistical difference in preference of time for exploring either object between EphB2 KO and WT mice. In the test sessions **(B,D)**, the cognitive index of the EphB2 KO mice was significantly reduced when compared to WT groups both at 1 and at 24 h after training. *P < 0.05, vs. the wild type group. **(E)** Schematics of the novel object recognition and novel object location tests.

Human spatial cognitive performance is not usually tested with strong stressors, such as water immersion in a Morris water maze. Therefore, other non-stressing behavioral tests, such as novel object location and novel object recognition tests, were performed in the present study as shown in Figure 3E. In the training session, statistical analyses revealed no differences in preference for either object based on the ratio of time spent on exploring either of them and total exploring time between WT and KO groups (Figures 3A,C). Strikingly, during the test session, the discrimination index of EphB2 KO mice was significantly reduced when compared to WT mice both at 1 h and at 24 h after training in ORT and OLT (*p's* < 0.05; Figures 3B,D). Thus, the time spent exploring the novel object or the displaced one by the WT group was longer than that of the familiar object or non-displaced one. In contrast, EphB2 KO mice spent less time to explore the novel object or novel-location object.

### Reduced hippocampal neural progenitor cells in EphB2 KO mice

**Figure 4:**
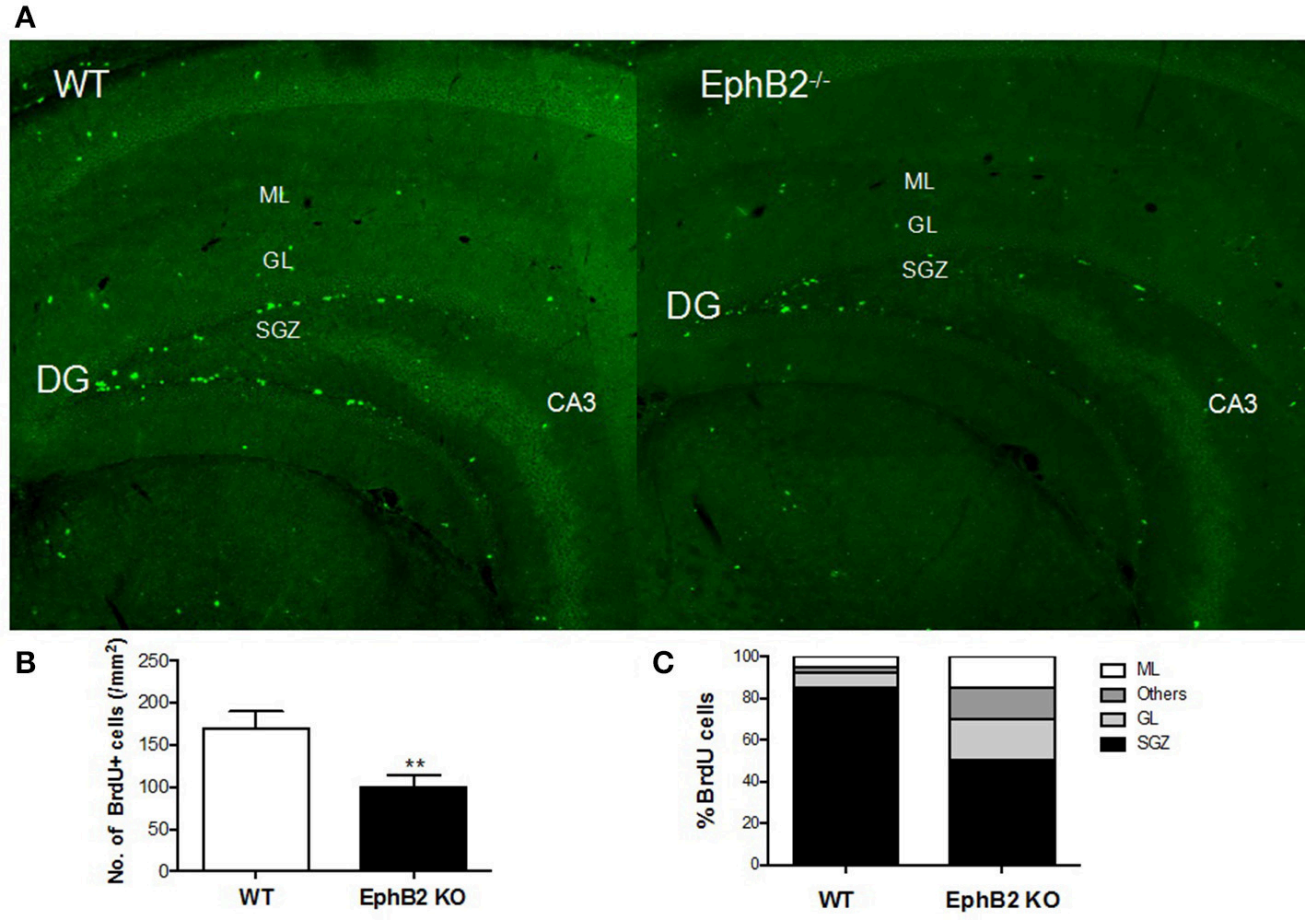
Hippocampal neuronal progenitor cells were reduced in EphB2 KO mice. **(A)** Immunofluorescence image of hippocampal slices in mice. **(B)** The number of BrdU+ cells per mm^2^ in the subgranular zone (SGZ) of dentate gyrus (DG) in EphB2 KO and WT mice. **(C)** The distribution of BrdU-labeled cells in the molecular layer (ML), granule cell layer (GL), SGZ and the left area of dentate gyrus in EphB2 KO and WT mice. **P < 0.01, vs. the wild type group.

While EphB2 has been shown to be involved in hippocampal neurogenesis, it remained unclear whether EphB2 inactivation impaired neurogenesis in the adult hippocampus. The present study firstly investigated BrdU-labeled cells (BrdU-positive cells or BrdU+ cells) in the dentate gyrus of the hippocampus 24 h and 28 days, respectively, after BrdU injection in EphB2 KO and WT mice. BrdU-labeled cells were found all around the dentate gyrus of hippocampus both in the EphB2 KO mice and WT littermates (Figure 4A). The number of BrdU+ cells in subgranular zone (SGZ) was 185.1 ± 26.9 counts/mm^2^ in the dentate gyrus (DG) of WT mice. There was a marked reduction in the number of BrdU+ cells in SGZ in the EphB2 KO mice (*p* < 0.01; Figure 4B). Not only the number, but also the distribution of BrdU+ cells in the dentate gyrus were very different between WT and EphB2 KO mice. Although in EphB2 KO mice the majority of BrdU+ cells were found in the SGZ, the BrdU+ cells were not confined to the SGZ. They were scattered throughout the entire dentate gyrus instead, including in the molecular layer (ML), the granule cell layer (GL) and the hilus (ML). The total numbers of BrdU+ cells in EphB2 KO mice were significantly decreased in the SGZ, where their numbers decreased by almost half when compared to those of WT littermates (*p* < 0.01; Figure 4C). However, the differences of BrdU+ cells between WT and EphB2 KO mice did not achieve significance in the GL and ML subregions (Figure 4C).

**Figure 5:**
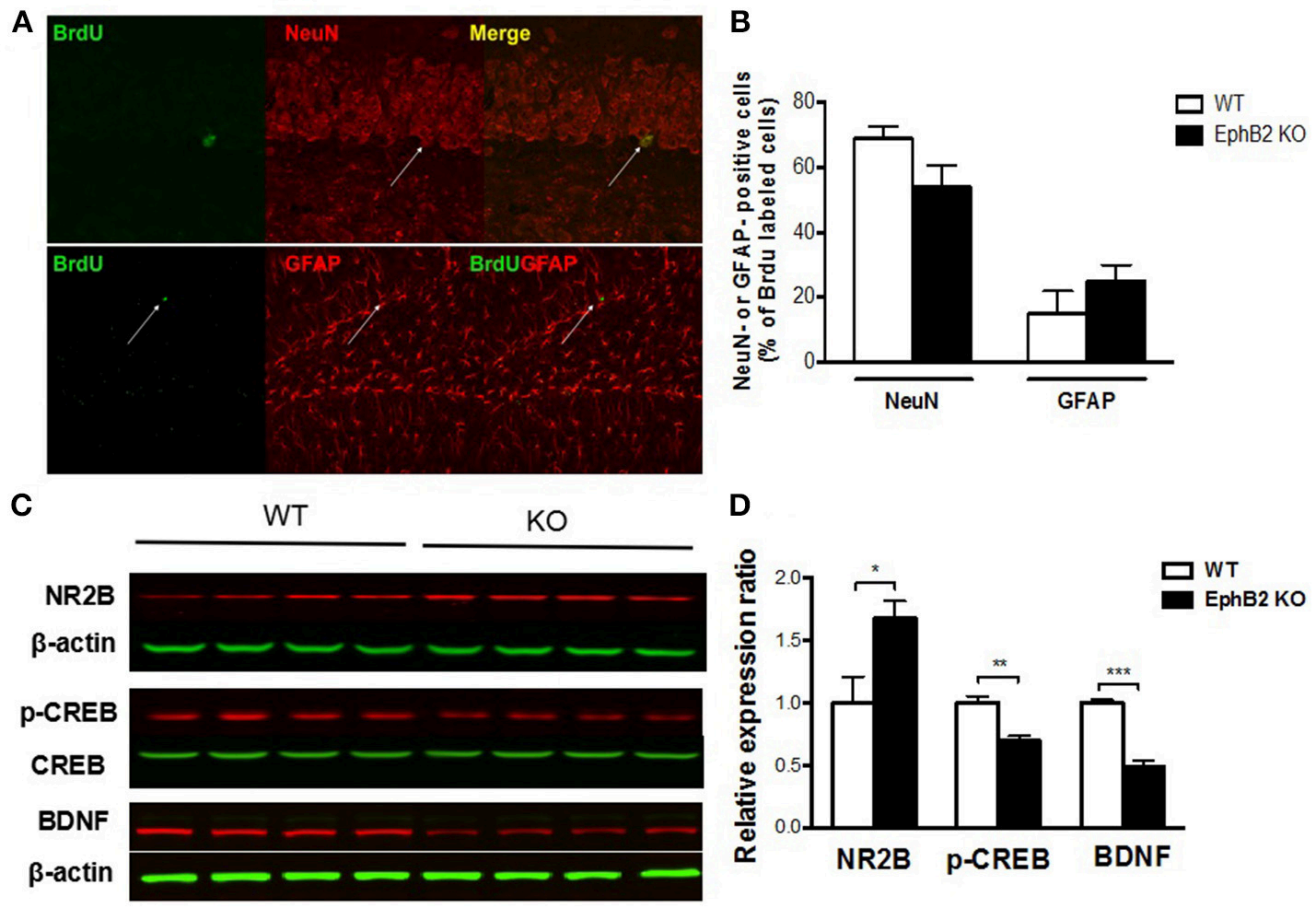
Hippocampal neuronal progenitors differentiated into adult neurons and glias in EphB2 KO mice **(A,B)**, which were related to altered NMDA receptor 2B (NR2B), pCREB and BDNF levels **(C,D)**. **(A)** Double immunostaining for BrdU and NeuN or GFAP of mouse hippocampal slices. **(B)** The composition of BrdU labeled cells in EphB2 KO mice and the WT littermates. **(C,D)** NR2B was increased, while pCREB and BDNF were decreases in the EphB2 KO mice. *P < 0.05, **P < 0.01, and ***P < 0.001, vs. the wild type group.

Consequently, we determined whether cells born in the SGZ of the adult rodent hippocampus express markers of adult neurons or of glias as they differentiate and mature. Mice were sacrificed 4 weeks after injection of BrdU, and double immunostaining of brain tissue was performed for BrdU and either for NeuN, a marker of mature neurons to assess neurogenesis, or for GFAP, a marker for stem cells and reactive astrocytes to assess gliogenesis. The results showed that the majority of BrdU+ cells were neurons (71 ± 2%) and not astrocytes (16 ± 3%) (Figures 5A,B) in wild type controls. Although a decrease in the proportion of mature neurons in EphB2 KO mice (55% in EphB2 KO vs. 71% in WT) and an increase in the proportion of glial cells (25% in EphB2 KO vs. 16% in WT) were found, the changes did not reach statistical significance. These results indicate that the EphB2 inactivation did not significantly affect the differentiation of cells into neurons or glia.

### EphB2 deficiency altered NMDA receptor 2B, pCREB, and BDNF expression in the hippocampus

To explore the molecular mechanism of EphB2 mediated hippocampus-dependent emotional and memory function, we determined whether EphB2-controlled NR2B receptor related neurotrophic signaling in the hippocampus. The protein level of surface NR2B receptor subunit was increased in EphB2 KO mice compared to WT mice (*p* < 0.05; Figures 5C,D), which indicates EphB2 may modulate NR2B function and localization in a subunit-specific manner. We then examined whether EphB2 inactivation changes the expression or activity status of proteins that are downstream of the NMDA receptor 2B. The protein levels of phospho-CREB (pCREB) and of BDNF were investigated. We found that the pCREB level in the hippocampus of EphB2 KO mice was decreased when compared to that of WT littermates (*p* < 0.01), though the total CREB did not change. The BDNF expression was also decreased in EphB2 KO mice when compared to WT littermates (*p* < 0.001).

### NMDA receptor 2B mediates EphB2 deficiency-induced depression-like behaviors and memory impairment

**Figure 6:**
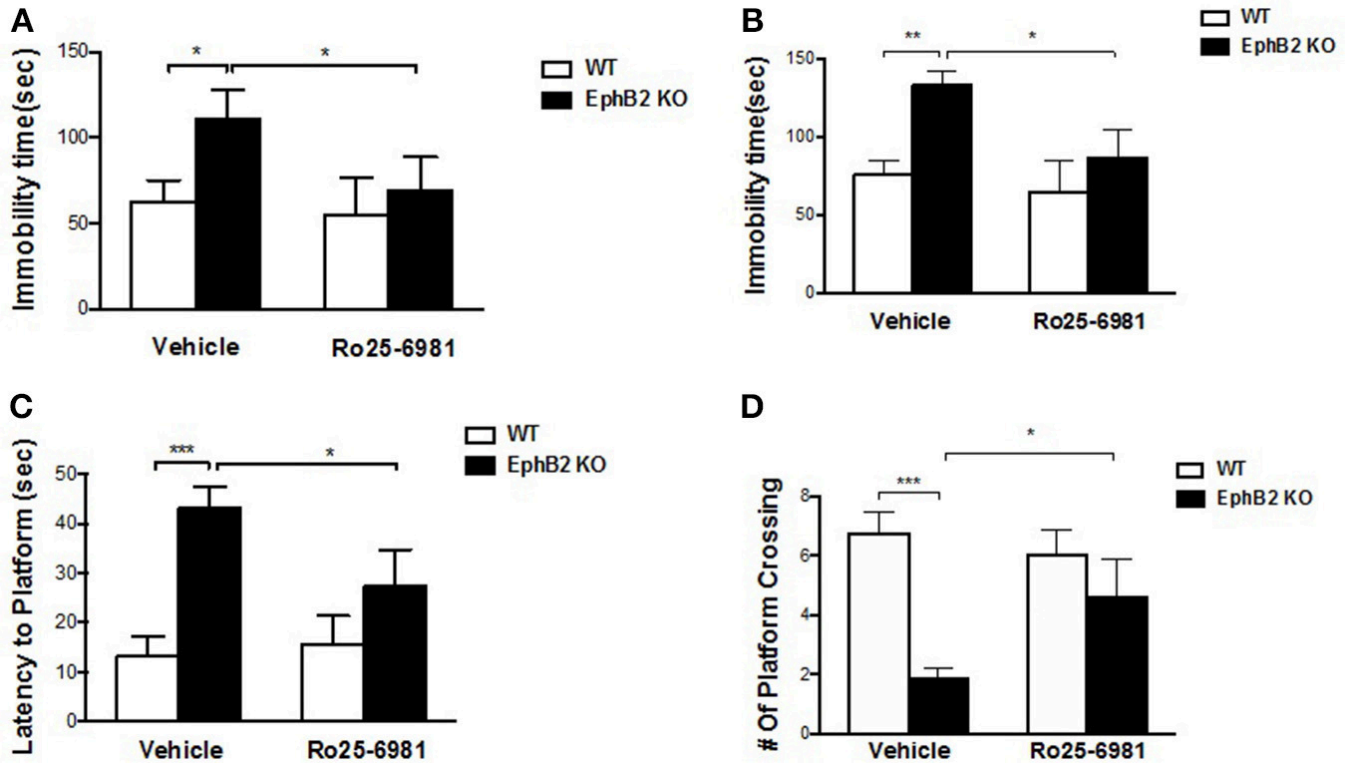
NMDA receptor 2B mediated EphB2 deficiency-induced depression-like behaviors and memory impairment. Ro25-6981 reversed EphB2 deficiency-induced increased immobility in the forced swimming **(A)** and tail suspension tests **(B)**. Ro25-6981 also reversed EphB2 deficiency-induced increased latency to the platform and decreased crossing numbers in the probe trials **(C,D)**. *P < 0.05, **P < 0.01, and ***P < 0.001, vs. the wild type group.

**Figure 7:**
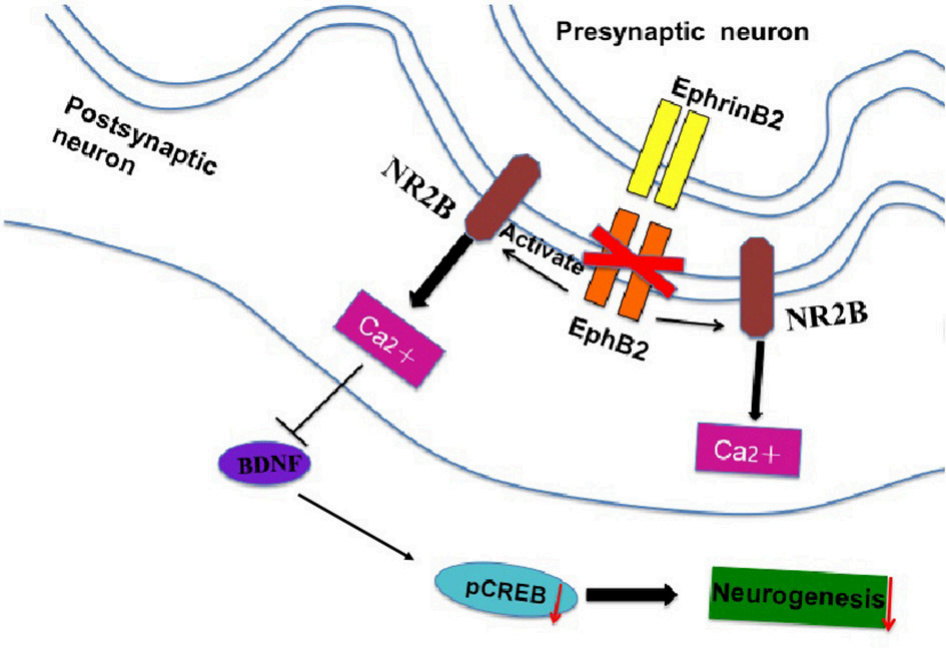
The signal cascade diagram summarizes how EphB2 deficiency in the postsynaptic membrane of neurons may induce behavioral and cellular changes. In EphB2 KO mice, NR2B expression is activated, which blocks the neuroprotective effects of EphB2 and subsequent neurogenesis in the hippocampus.

NMDA receptor 2B antagonist Ro25-6981 significantly prevented depression-like bevhaviors in EphB2 KO mice (Figures 6A,B), as evidenced by reducing the immobility time by 34 and 37% in the forced swimming and tail suspension tests, respectively (*p's* < 0.05). The locomotor activity did not change after treatment of EphB2 mice and WT littermates with Ro25-6981 (data not shown). In the probe trails of the Morris water maze test, Ro25-6981 prevented the EphB2-induced increase of the latency to reach the platform (*p* < 0.05; Figure 6C). The decreased number of crossings to the target quadrant of EphB2 mice was also prevented 30 min after treatment with Ro25-6981 in the test session (*p* < 0.05; Figure 6D). These effects of Ro25-6981 on EphB2 KO mice indicate that NR2B directly mediated the behavioral abnormalities induced by EphB2 inactivation. The EphB2-mediated behavioral, cellular and molecular signaling changes are summarized in Figure 7 and highlight the central signaling role of the EphB2 receptor.

## Discussion

Here we show that EphB2 plays an important role in depression-like behaviors, cognitive and memory impairment by inactivating the gene using a knockout approach. We found EphB2 KO mice, in which the EphB2 gene is inactivated, have depression-like behaviors, as evidenced by increased immobility time both in the tail suspension and forced swimming tests. The significant memory loss and cognitive impairment of these mutants were also observed in Morris water maze, novel object recognition and novel object location tests. EphB2 inactivation also induced a decrease in hippocampal neurogenesis and alterations in NR2B, pCREB and BDNF expression. However, these EphB2 KO related behavioral abnormalityies were prevented by NMDA receptor 2B antagonist Ro25-6981. These results demonstrated for the first time that EphB2-NR2B forward signaling contributes to controlling depression and cognitive disorders.

A common structural arrangement of Eph receptors has an extracellular ligand-binding domain, a transmembrane domain and an intracellular domain with multiple docking sites for downstream signaling molecules. Previous studies linked EphB2 to the pathology of anxiety and to memory processes since the lack of EphB2 in germline-targeted mice replicated deficiencies in long-term potentiation (LTP) and long-term depression (LTD), as well as behavioral impairments (Grunwald et al., 2001). To better understand how EphB2 mutation causes depression and the cognitive impairment, we used a mouse model engineered to have an inactivating mutation in the entire EphB2 receptor (Henkemeyer et al., 2003). Our findings show that inactivation of EphB2 results in depression-like behaviors in mouse model of despair tests, such as an increase in immobility time in forced swimming and tail suspension tests. The locomotor activity did not differ between wild type and EphB2 knockout mice, which ruled out the false positive or negative results regarding depressive behaviors. These results are in agreement with and extend the results of previously published, which suggested that overexpression of EphB2 rescued depression- and anxiety-like behaviors in chronically stressed mice (Zhang et al., 2016). Indeed, clinical trials found that major depressive disorder often includes depressed mood and cognitive disturbances; thereby enhancing learning and memory in these patients predicts improvement of depressive disorder symptoms (Jaeger et al., 2006). Following recognition of EphB2 knockout-induced depression-like behaviors, learning and memory deficits induced by EphB2 knockout were also found in the water maze test, as mice showed longer latency to catch the platform during training blocks. In probe trials, EphB2 KO mice showed a longer latency to arrive in the previous platform area and fewer crossings over the previous platform location, both at and 24 h after training, indicating a predominant role of EphB2 in mediating acquisition and memory processes. In addition, we extended the study to cognitive performance and found that EphB2 inactivation altered object recognition and spatial memory (location) in both novel object recognition and novel object location tests, as shown by the decreased time to explore the novel object and novel location. These data are consistent with previous studies that suggested early deficiency of EphB2 receptor preceded the onset of memory decline, while conversely overexpression of EphB2 rescued impaired cognition by regulating synaptic plasticity (Cissé et al., 2011; Hu et al., 2017). Our results extend these findings for the first time and suggested that deficiency of EphB2 was responsible for both depression-like behaviors and for related learning and memory deficits.

The neurogenesis in subgranular zone (SGZ) of the dentate gyrus involves a complex process including cellular migration and interpretation and processing of information. Early stage neural progenitors in the adult hippocampus migrate a short distance into the granule cell layer (GL) and terminally differentiate mainly into new dentate granule neurons. These newly generated granule neurons elaborate thick dendritic branches into the molecular layer (ML) of the dentate gyrus and begin receiving functional synaptic inputs from the entorhinal cortex and local inhibitory neurons while extending axons into hilus of the CA3 region through a mossy fiber pathway to complete integration into the existing neural circuitry (Laplagne et al., 2006; Hu et al., 2008). As the basic process of neuron generation, neurogenesis may preserve cognitive functions and aid in regulating emotional responses throughout life. Impaired neurogenesis in the subgranular zone of the dentate gyrus in adult mice brain may account for the pathogenesis of depression, defective contextual fear memory and spatial memory (Gheusi et al., 2000; Clelland et al., 2009). The functions of EphB2 in stem cells and progenitor cells of the dentate gyrus have not previously been described, even though EphB2/ephrinB signaling has been shown to increase cell proliferation in the SGZ and subventricular zone of normal rats (Xing et al., 2008). To understand the effects of EphB2 inactivation on neurogenesis, we evaluated progenitor proliferation and differentiation by labeling dividing newborn cells with the thymidine analog BrdU. We observed that the number of BrdU+ cells decreases significantly in the SGZ subregion in EphB2 KO mice, even though they appeared to scatter around the entire dentate gyrus including SGZ, GL, ML, and Hilus. Notably, 70–75% of BrdU+ cells differentiate and mature into neurons expressing the neuronal marker NeuN and 20% of them differentiate into glia expressing the glial marker GFAP in the normal animals based on the previous study (Xu et al., 2007), which were consistent with our findings. The present data indicated that nearly 55% of BrdU-positive cells matured into neurons and 25% to glia in EphB2 KO mice, which suggested a lower percentage of matured neurons compared to WT littermates. Further statistical analysis found a decreasing trend in the number of BrdU/NeuN-double stained neurons in EphB2 KO mice; while a tendency to increase the glial cell numbers was also observed in EphB2 KO mice. The remaining 20% of cells were not labeled with either neuronal or astrocyte markers; these cells might represent a phenotype not labeled, or may be located deeper in the tissue section and therefore were not accessible to the antibodies used. Our results suggested that complete loss of EphB2 receptor leads to a disruption of the normal program of neurogenesis in the adult hippocampus.

The progenitors of neuronal cells localize NMDA receptors 2A/2B on their surface, which facilitate glutamate-mediated axonal growth cone development and neuron migration (Englund et al., 2005; Iulianella et al., 2008; Chakraborty et al., 2017). The synaptic localization of the NR2B receptor plays a major role in the process of terminal differentiation of the neural progenitors. The EphB2 receptor is critical for embryonic and postnatal development of the dentate gyrus and for the modulation of neuronal networks (Behar et al., 1999; Catchpole and Henkemeyer, 2011). It mediates tyrosine phosphorylation of NR2B and it stabilizes NMDA receptors on the cell surface, thereby improving the response of NMDA receptors (Salter and Kalia, 2004). However, questions remain pertaining to how EphB2 mediates neurogenesis by interacting with NR2B for subsequent signal transduction. The correlation between EphB2 and NMDAR1/NMDAR2 requires ephrin-induced EphB2 oligomerization instead of EphB2 kinase activity, indicating that the extracellular domain of EphB2 is involved in this interaction (Henderson et al., 2001). We therefore directly examined the role of EphB2 in glutamate-related neuroprotection by measuring whether EphB2 inactivation changes NR2B levels and the expression of its downstream proteins in the hippocampus. Our study shows that mice lacking the entire EphB2 receptor have a significant increase in NR2B expression in the hippocampus. These findings differ in part from recently published results suggesting that overexpression of EphB2 in hippocampus rescues behavioral deficits and increases the expression of NR2B (Zhang et al., 2016; Hu et al., 2017). It is possible that the mutation of EphB2 triggers the dissociation of NMDAR in the hippocampus, resulting in abnormal expression of NR2B and decreased neurogenesis. The subsequently decreased pCREB and BDNF expression in the hippocampus of EphB2 KO mice supports the hypothesis that the downstream molecules genetically interact with EphB2 to regulate neurogenesis and suppress depression-like behaviors and cognitive deficits. The fact that NR2B antagonist prevented depression-like behaviors and cognitive impairment in EphB2 KO mice further confirms the involvement of NMDA receptor 2B in EphB2 mediated behavioral abnormalities.

In conclusion, our findings show that inactivating EphB2 produces depression-like behaviors and deficits in memory and cognitive processes. These changes may be related to the cellular and molecular changes we detected: decrease in hippocampal neurogenesis and abnormalities in signaling molecules such as NMDA receptor 2B and its downstream pCREB and BDNF molecules. Understanding the role of EphB2 in depression and cognitive processes may provide novel molecular intervention targets and enable future treatment of major depressive disorder.

## Author contributions

LZ conceived and wrote the manuscript. VL, TS, GL, and ZL performed the experiments. YX and VL revised the manuscript and XZ and VL jointly conceived and directed the manuscript.

### Conflict of interest statement

The authors declare that the research was conducted in the absence of any commercial or financial relationships that could be construed as a potential conflict of interest.
